# Caesarean Section in Peru: Analysis of Trends Using the Robson Classification System

**DOI:** 10.1371/journal.pone.0148138

**Published:** 2016-02-03

**Authors:** Vilma Tapia, Ana Pilar Betran, Gustavo F. Gonzales

**Affiliations:** 1 Instituto de Investigaciones de la Altura, Universidad Peruana Cayetano Heredia, Lima, Peru; 2 UNDP/UNFPA/UNICEF/WHO/World Bank Special Programme of Research, Development and Research Training in Human Reproduction, Department of Reproductive Health and Research, World Health Organization, Geneva, Switzerland; 3 Department of Biological and Physiological Sciences, Faculty of Sciences and Philosophy, Universidad Peruana Cayetano Heredia, Lima, Peru; Hospital de Especialidades del Niño y la Mujer de Queretaro, MEXICO

## Abstract

**Introduction:**

Cesarean section rates continue to increase worldwide while the reasons appear to be multiple, complex and, in many cases, country specific. Over the last decades, several classification systems for caesarean section have been created and proposed to monitor and compare caesarean section rates in a standardized, reliable, consistent and action-oriented manner with the aim to understand the drivers and contributors of this trend. The aims of the present study were to conduct an analysis in the three Peruvian geographical regions to assess levels and trends of delivery by caesarean section using the Robson classification for caesarean section, identify the groups of women with highest caesarean section rates and assess variation of maternal and perinatal outcomes according to caesarean section levels in each group over time.

**Material and Methods:**

Data from 549,681 pregnant women included in the Peruvian Perinatal Information System database from 43 maternal facilities in three Peruvian geographical regions from 2000 and 2010 were studied. The data were analyzed using the Robson classification and women were studied in the ten groups in the classification. Cochran-Armitage test was used to evaluate time trends in the rates of caesarean section rates and; logistic regression was used to evaluate risk for each classification.

**Results:**

The caesarean section rate was 27% and a yearly increase in the overall caesarean section rates from 2000 to 2010 from 23.5% to 30% (time trend p<0.001) was observed. Robson groups 1, 3 (nulliparous and multiparas, respectively, with a single cephalic term pregnancy in spontaneous labour), 5 (multiparas with a previous uterine scar with a single, cephalic, term pregnancy) and 7 (multiparas with a single breech pregnancy with or without previous scars) showed an increase in the caesarean section rates over time. Robson groups 1 and 3 were significantly associated with stillbirths (OR 1.43, CI95% 1.17–1.72; OR 3.53, CI95% 2.95–4.2) and maternal mortality (OR 3.39, CI95% 1.59–7.22; OR 8.05, CI95% 3.34–19.41).

**Discussion:**

The caesarean section rates increased in the last years as result of increased CS in groups with spontaneous labor and in-group of multiparas with a scarred uterus. Women included in groups 1 y 3 were associated to maternal perinatal complications. Women with previous cesarean section constitute the most important determinant of overall cesarean section rates. The use of Robson classification becomes an useful tool for monitoring cesarean section in low human development index countries.

## Introduction

Cesarean section (CS) rates continue to increase worldwide [1, 2], in both high-income and low-income countries. CS rates in Latin America including Peru rose over the period 2000–2010 [3] and represent the region with highest increases [4]. The worldwide rise in CS is a major public health concern and cause of considerable debate due to potential maternal and perinatal risks, cost issues and inequity in access [3, 5]. An increase in the use of CS particularly in the public sector and in low-resource settings may notably affect health services by increased rates of maternal/neonatal complications [6] but also in economic terms [7].

The main determinants of this disparity and specific reasons for the increase in CS rates in most of the world remain unclear. In order to propose and implement effective measures to reduce or increase CS rates where necessary, first, it is essential to identify what groups of women are undergoing CS and second, investigate the underlying reasons for trends. However, monitoring CS rates in a reliable and comparable manner continues to be an unmet need. Over the last decades, several CS classification systems have been created and proposed for different purposes. In 2011, a systematic review of available classifications concluded that the Robson (also known as the 10-group) classification is in the best position to fulfill current international and local needs, and that efforts to develop an internationally applicable CS classification would be most appropriately placed in building upon this classification [5].

In 2015, WHO proposed the Robson classification system as a global standard for assessing, monitoring and comparing CS rates [8]. This system classifies women into one of ten categories that are mutually exclusive but totally inclusive that are based on five obstetric characteristics that are routinely collected in health facilities: 1) parity (nulliparous, multiparous with and without previous caesarean section), 2) onset of labor (spontaneous, induced or pre-labour caesarean section), 3) gestational age (preterm or term), 4) fetal presentation (cephalic, breech or transverse) and 5) number of fetuses (one or more than one) [9]. The classification is simple, robust, reproducible, clinically relevant and prospective and thus, every woman admitted for delivery can be immediately classified into one of the ten groups based on these few basic characteristics [9, 10].

The Robson classification has gained recognition over the last decade and increasing number of facilities and countries are using it to understand and monitor their CS rates. [10] However, we are not aware of any analysis using the Robson classification in Peru, country which has more than doubled its CS rate at national level in 12 years from 12.7% in 2000 to 26.5% in 2012 (www.dhsmeasure.com), and it is one of the 75 countries prioritized in the *Countdown to 2015 for Maternal*, *Newborn and Child Survival* (so-called Countdown) to track and stimulate progress toward targets in MDG 4 and MDG 5 [11].

In public hospitals in Peru, data on pregnancy and delivery are recorded in the Perinatal Information System, a database maintained by Ministry of Health. The present study aimed to apply the Robson classification to this Peruvian database, to analyze trends in CS rates over a period between 2000 and 2010 and to identify the groups of women who, according to the Robson classification, are the major contributors to the increasing rates. In addition, we will assess variation of maternal and perinatal outcomes over time by Robson group and we expect the findings of this analysis will be able to inform the design and focus of strategies targeted to optimize the use of CS in Peru.

## Materials and Methods

### Setting, design and source of data

For this analysis, we included all women and their newborns from 43 health facilities in Peru included in the Latin American Perinatal Information System between 2000 and 2010. The Perinatal Information System contains information collected prospectively from time of presentation at the facility until the second or fifth day post-partum (vaginal or cesarean delivery). Data were obtained from the SIPs (Information Perinatal Systems) of Peruvian public hospitals following approval from the Peruvian Vice Minister of Health. This data cannot be made publicly available. Other researchers interested in obtaining these data should contact Dr. Ernesto Gozzer, Chief of the National Institute of Health in Peru (egozzer@ins.gob.pe).

Around 12% of the deliveries in Peru [12] occur in these 43 Public facilities that are located in the main town of each geographic subdivision of the country named department. These facilities are distributed among the three geographical regions in Peru, the coast, altitude and jungle. Altitude is considered when place of pregnancy and birth was over 2000 m above sea level.From the first antenatal visit until discharge of both mother and neonate, the attendant physicians or midwifes collect data in the perinatal clinical record in check-box format, which included demographic information, reproductive history, maternal characteristics, prenatal care, labor management, maternal complications during pregnancy, delivery, and the puerperium, and neonatal outcomes. Data are then entered into computer records and verified for quality control at each site.

Patient records/information was anonymized and de-identified prior to analysis and prior receiving the database in our hands.

### Variables

**Fig 1:**
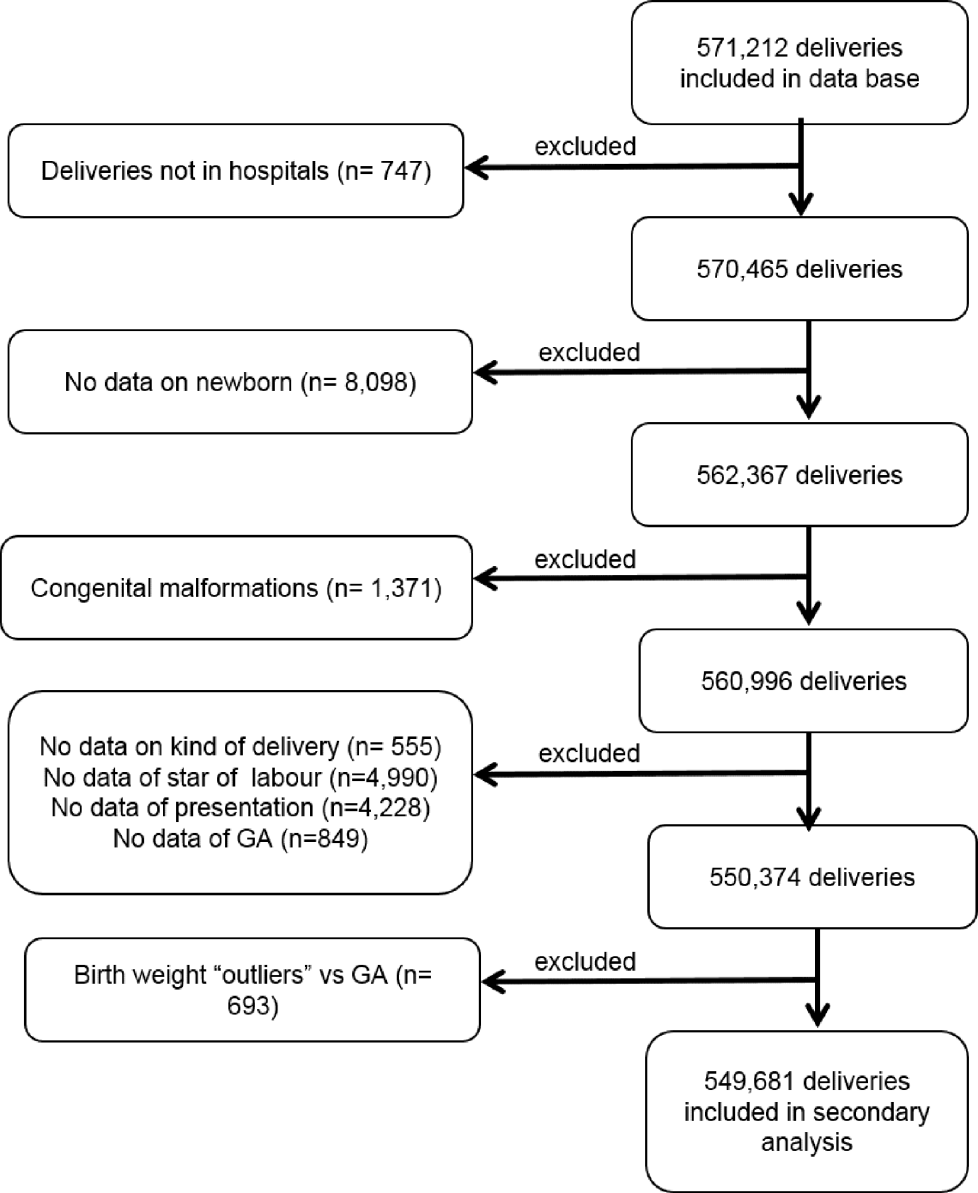
Flow chart of the selection of study participants of the 549,681 deliveries assessed in the secondary analysis.

All the variables necessary for the application of the Robson classification were available in the Perinatal Information System. The classification was constructed according to the proposed methodology [9, 2, 13–14]. The ten-group classification system is presented in Table 1. Women who could not be classified due to missing data in at least one of the variables of the Robson classification were excluded: start of labor 0.80% (4,990), presentation 0.75% (4,228) and GA 0.15% (849). Including all excludes cases (Fig 1), the total percentage of excluded represented 9.62% of the initial sample (21,531 out of 571,212).

**Table 1:** The Ten Group Classification System.

Robson Group	Characteristics
1	Nulliparas; single cephalic term pregnancy; spontaneous labor
2*a	Nulliparas; single cephalic term pregnancy; induced labor
2*b	Nulliparas; single cephalic term pregnancy; planned cesarean delivery
3	Multiparas without uterine scar; single cephalic term pregnancy; spontaneous labor
4*a	Multiparas without uterine scar; single cephalic term pregnancy; induced labor
4*b	Multiparas without uterine scar; single cephalic term pregnancy; planned cesarean delivery
5	Multiparas with a scarred uterus; single cephalic term pregnancy
6	Nulliparas; single breech pregnancy
7	Multiparas; single breech pregnancy (including women with a scarred uterus)
8	All women with a multiple pregnancy (including women with a scarred uterus)
9	All women with a single oblique or transverse pregnancy (including women with a scarred uterus)
10	All women with a single cephalic preterm pregnancy (including women with a scarred uterus)

*Groups 2 and 4 are subdivided as a) related to induced labor and b) related to cesarean pre-labor.

The flowchart showing the selection of the study participants is shown in Fig 1.

Studied outcomes were stillbirth rate, low birth weight rate, incidence of preeclampsia, and maternal mortality. Stillbirths were defined as fetal deaths occurred after 20 weeks of gestation or with weights higher than 500 g. Low birth weight was defined if birth weight was below 2500 g. Other variables included were preterm birth (<37 weeks), very preterm birth (<32 weeks), newborn death, Apgar score min 1 of less than 7, Apgar score min 5 of less than 7, and need for resuscitation. Intrauterine growth restriction was defined as stunted growth of the fetus, causing his weight is below the 10^th^ percentile expected for gestational age. Preeclampsia was defined as the presence of pregnancy-induced hypertension (systolic pressure of ≥140 mm Hg and/or diastolic pressure of ≥90 mm Hg) and proteinuria (≥300 mg/24 h) after 20 weeks of gestation. For this study, maternal mortality included deaths during pregnancy, labor and delivery and those after delivery until discharge from the hospital. Readmissions for complications or death after discharge are not recorded in the SIP under the same record so they cannot be linked. Maternal mortality ratio (MMR) was defined as the number of maternal deaths per 100,000 live births.

### Statistical analysis

Data in each group of the Robson classification system are presented in percentage for year of birth in three groups namely, 2000–2004; 2005–2007; and 2008–2010. We calculated CS rates as percentage in relation to obstetric population in each Robson group. Absolute contribution is the proportion of CS in relation to the total obstetric population, and relative contribution is the proportion of CS in each Robson group related to the total number of CS. Demographic and obstetric characteristic were determined by year of birth stratified. The chi-square test was used to calculate differences between groups for data expressed in proportions, and the Holm-Bonferroni method was used to counteract the problem of multiple comparisons. The confidence interval (CI) was defined at 95% for each variable. The Cochran-Armitage trend test was used to assess time trends in the rates of CS, setting significance at 5%. Rates of CS of Robson classifications groups were estimated according to delivery characteristics, newborn, year of birth and adverse perinatal outcomes. Estimates of crude and adjusted odds ratios (OR and aOR) with 95% CI were computed as measures of association between the variables. The adverse outcomes assessed were stillbirths, low birthweight, preeclampsia and maternal mortality. Adjusted ORs were derived through logistic regression models controlled by preeclampsia, prenatal visits, altitude and year of birth.

The trends in indications for induction and indications for CS in relevant categories of women in the Robson classification system were calculated over time (2000–2004, 2005–2007, and 2008–2010), the difference was assessed with chi square test and to adjust for multiple comparisons was used Holm-Bonferroni method. We grouped the data in three time periods to be able to assess differences and trends more easily and at the same time to be able to use all the data available for the 11 years with more power.

Significance was defined at a value of P< 0.05 for all statistical analysis.

The Universidad Peruana Cayetano Heredia (Lima, Peru) institutional review board approved the protocol. Data were analyzed using Stata software (v 10; StataCorp LP, College Station, TX, USA).

## Results and Discussion

The database used for this analysis included data for 571,212 women and their newborns. A total of 11,464 were excluded because women who did not delivered in the hospital (n = 747), no data on the newborn was recorded (n = 8,098), congenital malformations (n = 1,371), data was missing for mode of delivery (n = 555), newborns characterized as birth weight outliers (birth weight higher than 3 standard deviations, n = 693), unable to classify because of missing one or more of Robson variables (n = 10,067), being the final sample size analyze 549,681 women.

Table 2 presents the characteristics of the population included in the analysis by period of time. Compared with earlier years, in the period 2008–2010 we observed less obstetric population in the coast, less educated women, less married women, more multipara women, less multiple gestations, less fetal mortality, higher BMI, more women with CS pre labor, less use of forceps/vacuum, more prenatal visits, more postpartum bleeding, less anemia during pregnancy, and more HIV infection. Malaria was lower at 2005–2010 period related to 2000–2004 (P<0.01). Gestational age between 32–36 weeks (P<0.01) and low birthweight (P<0.01) were higher in the group assessed in 2008–2010 than before (2000–2007).

**Table 2:** Characteristics of obstetric population (including all births, singleton and multiple, from 28+0 to 44+6 gestational weeks), 2000–2010, years stratified.

Characteristic		2000–2004	2005–2007	2008–2010
		n = 207,362	n = 189,807	n = 152,512
		n (%)	n (%)	n (%)
**Maternal age**	** **			
	< 20^c^	42,186 (20.3)	38,919 (20.5)	33,431 (21.9)
	20–24**	62,647 (30.2)	54,763 (28.8)	43,227 (28.3)
	25–29**	47,657 (22.9)	42,886 (22.5)	33,693 (22.1)
	30–34	31,758 (15.3)	29,456 (15.5)	23,351 (15.3)
	35+**	22,337 (10.8)	22,804 (12.0)	17,769 (11.7)
	Missing data^c^	777 (0.37)	979 (0.52)	1,041 (0.68)
Geographical region**	** **			
	Coast	108,037 (52.1)	82,751 (43.6)	49,906 (32.7)
	High altitude	52,848 (25.5)	71,817 (37.8)	61,221 (40.1)
	Jungle	46,477 (22.4)	35,239 (18.6)	41,385 (27.1)
Education**	** **			
	None^b^	2,869 (1.4)	2,973 (1.6)	2,065 (1.35)
	Primary	34,552 (16.7)	33,880 (17.8)	29,392 (19.3)
	Secundary	131,504 (63.4)	115,175 (60.7)	90,597 (59.4)
	University	36,727 (17.7)	36,248 (19.1)	28,614 (18.8)
	Missing data	1,710 (0.82)	1,531 (0.81)	1,844 (1.21)
Civil Status**	** **			
	Married	46,788 (22.6)	33,007 (17.4)	22,268 (14.6)
	Common marital	126,521 (61.0)	126,983 (66.9)	107,683(70.6)
	Single	31,622 (15.2)	27,716 (14.6)	20,343 (13.3)
	Other	487 (0.23)	268 (0.14)	194 (0.13)
	Missing data	1,944 (0.94)	1,833 (0.97)	2,024 (1.33)
Parity**	** **			
	Nulliparous	97,686 (47.1)	87,442 (46.1)	69,908 (45.8)
	Multiparas	109,676 (52.9)	102,365 (53.9)	82,604 (54.2)
**Multiple gestations** **	** **	3,543 (1.71)	3,063 (1.61)	2,215 (1.45)
**Birthweight (grams)**	** **			
** **	**< 2500**^a^	17,481 (8.43)	16,547 (8.72)	13,304 (8.72)
** **	**2500–3999**	179,167 (86.4)	163,621(86.2)	131,379(86.1)
** **	**4000+**	10,714 (5.17)	9,639 (5.08)	7,829 (5.13)
**Gestational age**	** **			
	28–31 weeks	2,603 (1.26)	2,282 (1.20)	1,905 (1.25)
	32–36 weeks**	15,860 (7.65)	15,521 (8.18)	12,673 (8.31)
	≥ 37 weeks	188,899 (91.1)	172,004 (90.6)	137,934(90.4)
**Total CS rate**	** **			
	Primary CS rate	38,719 (18.7)	439,528(20.8)	35,759 (22.1)
	Repeated CS rate**	12,335 (5.95)	12,218 (6.44)	11,193 (7.34)
**Perinatal mortality**	** **			
	Fetal mortality (≥28	2,465 (1.20)	2,040 (1.10)	1,513 (1.0)
	weeks)**			
	Early neonatal mortality	1,762 (0.85)	1,200 (0.63)	884 (0.58)
	(0–7 days)^a^			
**History**	** **			
	Previous CS**	17,821 (8.60)	17,087 (9.0)	15,266 (10.1)
	Previous Preterm birth^b^	2,341 (1.13)	1,772 (0.93)	1,753 (1.15)
	Previous PE/E^c^	1,358 (0.65)	1,149 (0.61)	1,117 (0.73)
**BMI**	** **			
	<18.5	6,646 (3.2)*	5,736 (3.02)	3,956 (2.60)*
	18.5–24.9	132,940(64.1)*	121,269 (63.8)	92,412 (60.6)*
	25–29.9	45,247 (21.8)*	42,527 (22.4)	37,829 (24.8)*
	≥ 30**	10,231 (4.93)	9,671 (5.10)	9,999 (6.56)
	Missing data	12,362 (5.92)	10,604 (5.59)	8,316 (5.45)
Onset of labour**	** **			
	Spontaneous	170,071 (82.0)	153,872 (81.1)	122,993 (80.6)
	Induced	8,375 (4.04)	7,137 (3.76)	4,923 (3.23)
	Pre-labour CS	28,916 (13.9)	28,798 (15.2)	24,596 (16.1)
	Forceps/vacuum (%)**	2,344 (1.12)	940 (0.49)	372 (0.24)
Number of prenatal visits**	** **			
	None	49,877 (24.0)	38,714 (20.4)	22,468 (14.7)
	1–5	34,485 (16.6)	26,699 (14.1)	23,998 (15.7)
	6 or more	123,000 (59.3)	124,394 (65.5)	106,046 (69.5)
**Morbidity in current**	** **			
**pregnancy or delivery**				
	PE/E**	9,186 (4.43)	7,260 (3.82)	6,266 (4.11)
	Postpartum bleeding**	992 (0.48)	1,003 (0.53)	993 (0.65)
	Anaemia**	43,619 (21.0)	30,139 (15.9)	23,435 (15.4)
	HIV**	225 (0.11)	294 (0.15)	336 (0.22)
	Malaria^a^	222 (0.11)	43 (0.02)	44 (0.03)

Chi square and Holms-Bonferroni Test

** p<0.01 between different groups.

* p<0.01 between groups sharing the same symbols.

a: p<0.01 (2000–2004) vs (2005–2007); (2000–2004) vs (2008–2010).

b: p<0.01 (2000–2004) vs (2005–2007); (2005–2007) vs(2008–2010).

c: p<0.01, (2000–2004) vs (2008–2010); (2005–2007) vs (2008–2010). Value of hemoglobin below 11 g/dl was used to define anaemia.

The CS rate varied from 24.6% in the period 2000–2004 to 27.35% in 2005–2007, up to 30.03% during the period 2008–2010. Primary CS increased over time (18.7%, 20.8%, 22.1% for 2000–2004; 2005–2007 and 2008–2010, respectively; P<0.05). Similarly, repeated CS increased over time from 5.95% to 6.44% and 7.34% for the three groups respectively (P<0.05).

**Fig 2:**
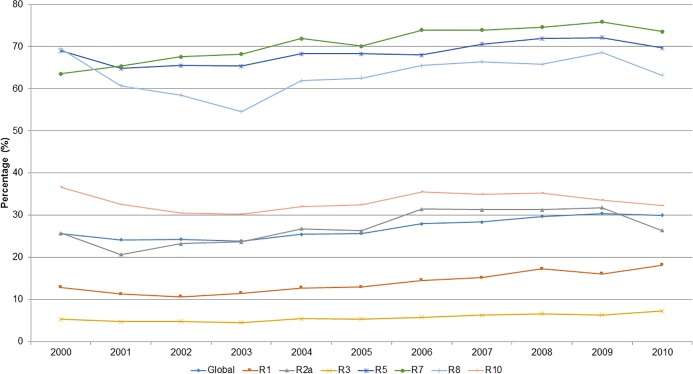
Evolution in cesarean sections (CS) rates in those groups of classifications of Robson with trend test p<0.01, between the years 2000 and 2010, in public hospital of Perú.

The yearly variation in the overall CS rates and by Robson group between 2000 and 2010 is displayed in Fig 2. Between 2003 and 2009 the overall CS rate increased from 23.7% to 30.4% (*p*<0.01). Similarly, groups 1, 2a, 3, 5, 7, 8 y 10 of the Robson classification system increased CS rates over time.

Data on CS rates for the years 2000–2004, 2005–2007 and 2008–2010 according Robson classification are presented in Table 3. CS rates of group 1 increased over time from 11.7% during 2000–2004 to 17.0% in 2008–2010 (*p*<0.001). Similarly, absolute and relative contributions also increased. CS rates and absolute contribution of group 2 raised over time. The main contributor between groups 2a and 2b to the overall CS rate was the group 2b. CS rates of group 2a increased over time from 24.1% in 2000–2004 to 29.9% in 2008–2010 (p<0.01). However, the absolute contribution was unchanged over time, and the relative contribution decreased in 2008–2010 compared to values between 2000 and 2007. CS rates and the relative contribution of group 2b did not change over time although the absolute contribution increased.

**Table 3:** Cesarean sections (CS) rates using the Robson´s classification in Peru, 2000–2010.

Robson Group	2000–2004				2005–2007				2008–2010			
	Obstetric Population	Cesarean Section	Absolute Contribution	Relative Contribution	Obstetric Population	Cesarean Section	Absolute Contribution	Relative Contribution	Obstetric Population	Cesarean Section	Absolute Contribution	Relative Contribution
	n (%)/CI95%	n (%)/CI95%	%/CI95%	%/CI95%	n (%)/CI95%	n (%)/CI95%	%/CI95%	%/CI95%	n (%)/CI95%	n (%)/CI95%	%/CI95%	%/CI95%
**1**	72581 (35.0)*	8524 (11.7)*	4.11*	16.7^a^	64382 (33.9)	9206 (14.3)	4.85	17.8	50998 (33.4)	8660 (17)	5.68	19.3
** **	34.3 34.7	11.5 12.0	4.02 4.19	16.4 17.0	33.6 35.1	14.0 14.6	4.75 4.95	17.4 18.12	33.1 33.6	16.6 17.3	5.56 5.79	18.9 19.6
**2**	12548 (6.05)*	9340 (74.4)*	4.5	18.3	11844 (6.24)	9377 (79.2)	4.94	18.12	10046 (6.59)	8375 (83.4)	5.49	18.6
** **	5.94 6.15	73.6 75.2	4.41 4.59	17.9 18.6	6.13 6.35	78.4 79.9	4.84 5.03	17.79 18.45	6.46 6.71	82.6 84.1	5.38 5.60	18.3 18.9
**2a**	4108 (1.98)*	989 (24.1)	0.47^a^	1.94^a^	3365 (1.77)	1002 (29.8)	0.53	1.89	2239 (1.47)	671 (29.9)	0.44	1.49
** **	1.92 2.04	22.8 25.4	0.44 0.50	1.82 2.05	1.71 1.83	28.2 31.3	0.49 0.56	1.78 2.02	1.41 1.53	28.1 31.9	0.41 0.47	1.38 1.60
**2b**	8440 (4.07)*	8351 (98.9)	4.03*	16.3^a^	8479 (4.47)	8375 (98.8)	4.41	15.9	7807 (5.12)	7704 (98.7)	5.05	16.4
** **	3.99 4.15	98.6 99.1	3.94 4.11	16.0 16.7	4.38 4.56	98.5 99.0	4.32 4.50	15.5 16.2	5.00 5.21	98.4 98.9	4.94 5.16	16.0 16.7
**3**	71430 (34.4)	3545 (5.0)*	1.70*	6.85^b^	65323 (34.4)	3808 (5.83)	2	7.35	51793 (33.9)	3458 (6.68)	2.26	7.69
** **	34.2 34.6	4.84 5.16	1.65 1.76	6.63 7.07	34.2 34.7	5.65 6.01	1.94 2.07	7.13 7.58	33.7 34.1	6.46 6.89	2.19 2.34	7.45 7.94
**4**	7807 (3.76)^a^	5040 (64.5)*	2.43	9.87*	7902 (4.16)	5470 (69.2)	2.88	10.6	5740 (3.76)	4027 (70.2)	2.64	8.95
** **	3.68 3.84	63.5 65.6	2.36 2.49	9.61 10.1	4.07 4.25	68.2 70.2	2.81 2.96	10.3 10.8	3.66 3.86	68.9 71.3	2.56 2.72	8.69 9.22
**4a**	2997 (1.45)^a^	305 (10.2)	0.15	0.59	2632 (1.39)	287 (10.9)	0.15	0.54	1943 (1.27)	274 (14.1)	0.18	0.58
** **	1.40 1.51	9.09 11.1	0.13 0.16	0.53 0.67	1.34 1.44	9.74 12.1	0.13 0.17	0.48 0.61	1.22 1.34	12.6 15.7	0.16 0.20	0.16 0.20
**4b**	4810 (2.32)*	4735 (98.4)	2.28*	9.27*	5270 (2.78)	5183 (98.3)	2.73	10	3797 (2.49)	3753 (98.8)	2.46	8.34
** **	2.26 2.39	98.0 98.8	2.2 2.35	8.90 9.53	2.71 2.85	98.0 98.7	2.65 2.80	9.76 10.3	2.41 2.56	98.4 99.1	2.38 2.54	8.09 8.61
**5**	14924 (7.20)*	9893 (66.3)^b^	4.76*	19.1^b^	14122 (7.44)	9.731 (68.9)	5.11	18.7	3797 (8.41)	9167 (71.5)	5.99	20.3
	7.08 7.30	65.4 67.0	4.66 4.85	18.7 19.4	7.32 7.55	68.1 69.6	5.01 5.21	18.4 19.1	8.27 8.55	70.8 72.4	5.87 6.11	19.9 20.7
**6**	3723 (1.8)	3245 (87.1)	1.56^c^	6.27*	3080 (1.62)	2703 (87.7)	1.42	5.22	2401 (1.57)	2080 (86.6)	1.36	4.62
** **	1.74 1.85	86.0 88.1	1.51 1.61	6.06 6.48	1.56 1.67	86.5 88.9	1.37 1.48	5.03 5.41	1.51 1.64	85.2 88.0	1.31 1.42	4.43 4.82
**7**	3861 (1.86)	2620 (67.8)^b^	1.26^c^	5.06	3610 (1.9)	2623 (72.7)	1.38	5.06	2922 (1.92)	2192 (75)	1.44	4.87
** **	1.81 1.92	66.3 69.3	1.21 1.31	4.87 5.25	1.84 1.97	71.2 74.1	1.33 1.43	4.88 5.26	1.85 1.99	73.4 76.6	1.38 1.50	4.68 5.08
**8**	3543 (1.71)*	2125 (60)	1.02	4.16*	3063 (1.61)	1985 (64.8)	1.04	3.83	2215 (1.45)	1472 (66.4)	0.96	3.27
** **	1.66 1.77	58.6 61.8	0.98 1.06	3.99 4.33	1.55–1.66	63.1 66.5	1.00 1.09	3.67 4.00	1.39 1.50	64.4 68.4	0.91 1.01	3.11 3.44
**9**	2045 (0.99)	96.4	0.95	3.86	1897 (1.0)	1836 (96.8)	0.96	3.54	1446 (0.95)	1405 (97.2)	0.92	3.12
** **	0.95 1.03	66.3 69.3	0.90 0.99	0.90 0.99	0.95 1.04	95.9 97.5	0.92 1.01	3.39 3.70	0.91 1.00	96.2 97.9	0.87 0.97	2.96 3.29
**10**	14900 (7.19)*	1972 (31.9)^c^	2.23^c^	9.22^d^	14584 (7.68)	5007 (34.3)	2.65	9.72	12125 (7.95)	4116 (34.0)	2.71	9.21
** **	7.8 7.30	31.1 32.6	2.22 2.36	8.97 9.48	7.56 7.87	33.5 35.0	2.58 2.72	9.47 9.98	7.82 8.08	33.1 34.8	2.63 2.79	8.94 9.48
	207,362	51,054		100.00	189,807	51,746		100.00	152,512	44,952		100

Absolute contributions mean the proportion of cesarean sections in relation to the total population. Relative contributions represent proportion of each Robson group according to total number of cesarean rates. Test Chi square, test Holms-Bonferroni

* p<0.01 between all

a: p<0.01 2000–2004 vs 2005–2007 and 2005–2007 vs 2008–2010

b <0.01 2000–2004 vs 2008–2010

c p<0.01 2000–2004 vs 2005–2007 and 2000–2004 vs 2008–2010

d: p<0.01 2004–2007 vs 2008–2010.

CS rates of group 3 increased from 5% in 2000–2004 to 6.7% in 2008–2010 (p<0.01). Similarly, absolute and relative contributions also increased. CS rates and the absolute contribution of group 4 increased up to 2005–2007 (p<0.05) and it remained unchanged during 2008–2010 (p>0.05). The relative contribution was reduced on 2008–2010. The main contributor between groups 4a and 4b to the overall CS rate was the group 4b. CS rates of group 4a increased during 2008–2010 with respect to 2000–2007. However, the absolute and the relative contributions did not changed from 2000 to 2010. CS rates of group 4b did not change either. However, the absolute and the relative contributions increased during 2005–2007 but thereafter they were reduced.

CS rates of group 5 increased from 66.3% in 2000–2004 to 71.5% in 2008–2010 (p<0.0001). The absolute contribution increased from 2005–2007 to 2008–2010. In addition, the relative contribution increased in 2008–2010 respect to values in 2000–2007. CS rates of group 6 maintained unchanged over time although the absolute and relative contributions decreased. CS rates of group 7 increased from 67.6% in 2000–2004 to 75.0% in 2008–2010 (p<0.01). The absolute contribution increased in 2005–2010 respect to 2000–2004 but the relative contribution did not change.

CS rates of group 8 increased from 60.3% in 2000–2004 to 67.6% in 2008–2010 (p<0.01). The absolute contribution remained unchanged, however, the relative contribution decreased. CS rates and the absolute contribution of group 9 did not changed over time from 2000 to 2010 but the relative contribution decreased. CS rates and the absolute contribution of group 10 increased over time (p<0.01). However, the relative contribution remained unchanged.

The overall cesarean rate had the highest correlation with Robson group 1 (r = 0.93, p<0.01), followed by group 3 (r = 0.90, p<0.01), group 4a (r = 0.88, p<0.01) and group 7 (r = 0.84, p<0.01).

### Robson groups, selected labor characteristics, and maternal outcomes

Data on healthcare professional at delivery, use of forceps or vacuum, use of anesthesia, maternal mortality and post-partum hemorrhage by Robson group are presented in Table 4. Over 97% of women in groups 2b, 4b and 9 are attended by doctors. These figures remained unchanged between 2000 and 2010. Women in groups 5, 6 and 7 are attended by doctors in 77.8–92.5% of cases and similarly, remained unchanged over time.

**Table 4:** Trends in selected labor characteristics and maternal outcomes by Robson group.

Robson group	2000–2004						2005–2007						2008–2010					
	Attended by doctor	Attended by midwife	Instrumental delivery	Use of anesthesia	MMR	PPH	Attended by doctor	Attended by midwife	Instrumental delivery	Use of anesthesia	MMR	PPH	Attended by doctor	Attended by midwife	Instrumental delivery	Use of anesthesia	MMR	PPH
	%	%	%	%		%	%	%	%	%		%	%	%	%	%		%
1	33.2*	46.7*	1.76*	11.1*	10	0.51	30.3	58.6	0.89	11.9	20	0.44	28	70.3	0.46	14.9	20	0.6
2	83.4^a^	10.1*	1.31^a^	45.7^b^	60	0.60^b^	83.9	12.3	0.37	48.8	40	0.8	88.4	11.1	0.33	58.7	40	0.97
2a	51.3^a^	29.7*	3.77^a^	18.4^b^	0	1.39	46.4	41.9	1.16	18	0	1.1	51.5	47.1	1.21	28.1	40	2.1
2b	99	0.51	0.12	59.1^b^	80	0.2^c^	98.8	0.64	0.06	61	30	0.7	98.9	0.78	0.08	67.5	40	0.6
3	23.1*	55.3*	0.43*	4.65^b^	10	0.41	20.9	68.2	0.17	4.86	20	0.46	16.6	81.9	0.07	5.78	10	0.55
4	72.9	18.6*	0.45	38.3	60	0.53	74.3	20.8	0.11	39.5	100	0.78	76.4	22.9	0.12	49	30	0.94
4a	32.0^a^	47.4*	1.17^c^	9.78^b^	0	0.9	26.3	61	0.27	8.36	110	1.4	33.2	65.3	0.21	11.8	100	0.9
4b	98.5	0.77	0	56.1^b^	100	0.3^a^	98.3	0.74	0.04	55	90	0.5	98.5	1.2	0.08	68	0	0.9
5	77.9	13.5*	2.89*	45.9*	30	0.35^d^	77.2	19.6	1.03	50.3	30	0.51	77.2	22.4	0.34	56.9	20	0.6
6	92.5	6.0^b^	0.08	57.8^b^	0	0.20^d^	92.6	6.4	0.03	58.4	130	0.23	91.8	7.87	0.08	64.8	0	0.6
7	80.8	14.5	0.05	46.7^b^	130	0.41	82.6	16	0	49.9	220	0.7	83.8	15.7	0	57.3	30	0.55
8	82.4	13.2*	1.30*	44.4^b^	110	1.9	81.1	16.6	0.52	44.8	0	1.96	78.4	21	0.05	54.3	0	2.3
9	97.8	1.76	0.2	68.1	150	0.5	97.1	2.3	0.11	67	110	0.7	98	1.87	0.07	72	70	0.7
10	46.5^b^	38.8*	0.49*	20.5^b^	230	0.43^d^	46.5	47.6	0.21	21.5	190	0.6	42.6	56.5	0.07	24.1	210	0.73

MMR: maternal mortality ratio (per 100,000 live births). PPH: post partum hemorrhage. Test Chi square, Test Holms-Bonferroni

*: p<0.01 between all

a: p<0.01: 2000–2004 vs 2005–2007 and 2005–2007 vs2008–2010

b: p<0.01: 2000–2004 vs2008–2010 and 2005–2007 vs 2008–2010.

c: 2000–2004 vs 2005–2007 and 2000–2004 vs 2008–2010

d: p<0.01: 2000–2004 vs 2008–2010.

During 2000–2004, women from groups 1 (33.2%), 3 (23.1%) and 4a (32%) were attended by doctors. The percentages were reduced overtime in the groups 1 and 3 but not in 4a. Midwives ranging from 46.7% and 55.3% during 2000–2004 mainly attend women from groups 1, 4a and 3 and the rates in all three groups increased over time.

During 2008–2010, midwives attended 65.3–81.9% of the deliveries in these groups. Six out of twelve Robson groups have increased the attention by midwives. Namely the groups 1, 2a,3,4a, 8 and 10.

During 2000–2004, among all Robson groups, instrumental deliveries were most used in women of the groups 1, 2a, and 5, but assisted vaginal delivery decreased with time as observed for 2005–2007 and 2008–2010. Use of anesthesia was more frequent during 2000–2004 in the Robson groups 9 (68.1%), 2b (59.1%), 6 (57.8%) and 4b (56.1%). The values were significantly reduced overtime in the groups 2b, 4b and 6 but remained unchanged in the group 9.

### Robson groups and adverse outcomes

As expected, maternal mortality ratio was higher in groups 4b, 7, 8, 9 and 10 during 2000–2004. During 2004–2007, highest maternal mortality ratio was observed in the same groups except for group 8. During 2008–2010, maternal mortality was reduced in groups 7, 8 and 9. During 2000–2004 and during 2008–2010, the highest maternal mortality ratio was found in the group 10.

Post-partum hemorrhage was more prevalent in groups 8 (1.9%, 1.96% and 2.3% for 2000–2004, 2005–2007 and 2008–2010, respectively) and 2a (1.39%, 1.1% and 2.3%, respectively).

Higher rates of stillbirths during 2000–2004 were observed in the group 7, 9 and 10. The rates were significantly reduced over time up to 2008–2010. Rates for newborn deaths were higher during 2000–2004 in the groups 8 and 10, and these values were slightly reduced over time (Table 5).

**Table 5:** Trends in selected fetal and newborn outcomes by Robson group.

Robson Group	2000–2004							2005–2007							2008–2010						
	Stillbirth	Newborn death	LBW	Apgar 1' <7	Apgar 5' <7	IUGR	Need for resucitation	Stillbirth	Newborn death	LBW	Apgar 1' <7	Apgar 5' <7	IUGR	Need for resucitation	Stillbirth	Newborn death	LBW	Apgar 1' <7	Apgar 5' <7	IUGR	Need for resucitation
	%	n (%)	%	%	%	%	%	%	n (%)	%	%	%	%	%	%	n (%)	%	%	%	%	%
1	0.37	151 (0.21)^c^	3.52	6.23*	0.79	11.7	4.78	0.37	107 (0.17)	3.59	5.43	0.73	11.6	4.87	0.38	59 (0.12)	3.57	5.34	0.82	12.1	4.79
2	0.95	62 (0.49)^d^	4.73^d^	8.13	1.26	11.6	6.52	0.95	34 (0.29)	3.95	6.23	0.83	10.7	5.6	0.74	36 (0.36)	4.46	6.56	0.94	10.7	5.43
2a	1.7	13 (0.32)	2.97	8.14	0.8	8.62	7.62	1.52	3 (0.09)	3.09	7.63	0.83	9.39	7.73	2.05	6 (0.27)	3.75	7.2	0.98	9.47	8.13
2b	0.58	49 (0.58)	5.58	8.13^b^	1.48	13.0^b^	5.98	0.72	31 (0.37)	4.29	5.67	0.83	11.2	4.75	0.36	30 (0.38)	4.66	6.38	0.92	11.1	4.66
3	0.48	140 (0.20)	2.21	3.74^b^	0.47	7.14*	3.4	0.44	101 (0.15)	2.43	3.33	0.4	7.76	3.59	0.44	67 (0.13)	2.58	3.42	0.44	8.34	3.62
4	1.88	43 (0.55)	4.34	8.03	1.38	9.88	6.17	1.85	26 (0.33)	4.56	6.5	0.99	9.48	5.11	1.46	16 (0.28)	4.6	6.61	0.94	9.77	6.31
4a	2.47	8 (0.27)	2.8	6.03	0.8	6.91	6.31	3.04	3 (0.11)	3.27	6.09	0.61	7.26	6	2.47	3 (0.15)	2.88	5.12	0.41	7.62	7.77
4b	1.52	35 (0.73)	5.3	9.27^b^	1.75	11.7	6.09	1.25	23 (0.44)	5.2	6.7	1.18	10.6	4.67	0.95	13 (0.34)	5.48	7.36	1.21	10.9	5.56
5	0.49	35 (0.23)	2.18	5.14^b^	0.62	6.5	4.56	0.43	35 (0.25)	2.32	4.2	0.48	6.49	4.14	0.53	17 (0.13)	2.46	4.3	0.54	7.05	4.2
6	3.63	125 (3.36)^a^	7.04	18.4^a^	3.55	19.4	12.8	2.95	79 (2.56)	7.47	15.2	3.02	20.2	12.1	3.42	54 (2.25)	7.71	16.1	3.12	21.5	10.7
7	6.79^b^	142 (3.68)^b^	5.7	22.2	4.53	16	15.6	4.79	98 (2.71)	6.23	20.8	3.85	17	15.4	4.41	69 (2.36)	5.99	19.2	3.73	16.9	13
8	2.34	141 (3.98)	19.1	14.5	2.62	40.7	12.6	2.09	93 (3.04)	21.2	12.9	2.02	43	12	1.72	71 (3.21)	22.6	12.7	3.07	44.3	12.7
9	6.70^a^	62 (3.03)^c^	4.94	20.4	3.37	12.4	13.2	5.22	29 (1.53)	5.43	19.2	2.58	15.3	12.4	4.43	27 (1.87)	5.12	17.7	2.7	13.3	10.9
10	6.0*	861 (5.78)^b^	0	14.5	3.77	23.8	12.5	5.22	598 (4.10)	0	14.2	3.52	24.6	13.5	4.57	468 (3.8)	0	13.6	3.52	22.9	11.7

Apgar 1’: Apgar at the first minute, Apgar 5’: Apgar at the fifth minute. LBW: low birthweight (<2500 g). Test Chi square, Tests Holms-Bonferroni

*: p<0.01 between all

a: 2000–2004 vs 2008–2010

b:p<0.01: 2000–2004 vs 2005–2007 and 2000–2004 vs 2008–2010

c: p<0.01: 2000–2004 vs 2008–2010

d: p<0.01 2000–2004 vs 2005–2007.

Low birth weights were significantly higher in the groups 6, 7 and 8 during 2000–2004 with a trend to increase the rates with time. Intrauterine growth retardation (IUGR) was highest in the groups 8 and 10 with no change over time (Table 5). Highest rates of Apgar score<7 at first minute were observed in groups 7 (22.2%) and 9 (20.4%) during 2000–2004. These values are slightly reduced over time up to 19.2% and 17.7%, respectively for the period 2008–2010. Need for resuscitation was high in the groups 6 through 10 being highest in the group 7 (15.6%). All these figures in the last five groups of Robson showed a reduction trend over time. However, in the last period of time, the need for resuscitation continued to be high in these groups compared to the groups 1–5 (Table 5).

Increased ORs of stillbirths are observed with CS with respect to vaginal deliveries in the groups 1 and 3. Lower risks for stillbirths were observed in the groups 2a, 4a, 6, 7, 8, 9 and 10 by result of CS as compared with VD (Table 6). OR for LBW was higher associated with group 3 of Robson, and lower associated with groups 1 and 7 of Robson (Table 6). Preeclampsia was associated with CS in all Robson groups except group 4a (multiparous women without previous CS at term with single cephalic fetus in induced labor) (Table 6).

**Table 6:** Adjusted OR for stillbirths, low birth weight (LBW) and maternal mortality (MM) by Robson Group.

Robson Group	Stillbirths		Low birthweight		Pre eclampsia**		Maternal Mortality	
	OR*	IC 95%	OR*	IC 95%	OR	IC95%	OR*	IC 95%
1	1.43	1.175 1.727	0.84	0.778 0.904	3.88	3.663 4.101	3.39	1.592 7.223
2a	0.20	0.109 0.376	0.99	0.765 1.281	1.61	1.407 1.852	xxx	
2b	xxx		1.75	0.867 3.566	5.27	2.342 11.849	0.05	0.062 0.478
3	3.53	2.95 4.22	1.44	1.299 1.612	4.48	4.114 4.889	8.05	3.340 19.416
4a	0.49	0.272 0.885	1.10	0.732 1.670	1.09	0.852 1.399	12.32	1.984 76.513
4b	2.97	0.414 21.39	1.35	0.661 2.759	1.96	1.033 3.708	xxx	
5	0.76	0.570 1.014	1.12	0.971 1.293	2.34	2.053 2.674	3.72	0.467 29.773
6	0.03	0.024 0.043	0.65	0.531 0.809	2.51	1.567 4.013	xxx	
7	0.10	0.085 0.128	0.56	0.472 0.665	2.61	1.894 3.605	1.18	0.357 3.880
8	0.65	0.481 0.874	0.97	0.871 1.082	3.31	2.836 3.868	xxx	
9	0.13	0.089 0.182	0.83	0.456 1.528	3.49	0.859 14.245	xxx	
10	0.71	0.642 0.790	xxx		10.19	9.421 11.026	3.68	2.203 6.157

*Model adjusted for Preeclampsia, prenatal visits, altitude and year of birth.

**Model adjusted for prenatal visits, year of birth and altitude.

OR is compared with the reference value of 1 in the group with vaginal delivery.

Comparing the impact of CS respect to VD, higher ORs for maternal mortality were observed in groups 1, 3, 4a and 10, whereas an OR of 0.05 (0.006–0.478, IC95%) was observed in the group 2b (nulliparous at term with planned cesarean section) (Table 6).

The main indication for induction of labor was acute fetal distress (AFD). The indication for AFD was slightly reduced from 2000–2004 to 2005–2007, but thereafter was again increased in the Robson Group 4a and 10. The indication for pre-eclampsia was reduced overtime only in the group 2a (Table 7).

**Table 7:** Trends in indications for induction in the relevant categories of women in the Robson classification in Peru, 2000–2010.

Robson Group	2000–2004			2005–2007			2008–2010		
	Acute fetal distress	Cephalopelvic disproportion	Pre-eclampsia	Acute fetal distress	Cephalopelvic disproportion	Pre-eclampsia	Acute fetal distress	Cephalopelvic disproportion	Pre-eclampsia
	n (%)	n (%)	n (%)	n (%)	n (%)	n (%)	n (%)	n (%)	n (%)
2a	216 (5.2)	195 (4.7)	181(4.3)*	159 (4.6)	148 (4.3)	120 (3.5)	141 (6.25)	95 (4.21)	59 (2.62)
4a	93 (3.1)	46 (1.53)	54 (1.8)	62 (2.3)^a^	38 (1.4)	46 (1.7)	72 (3.68)	23 (1.18)	19 (0.97)
5	10 (4.4)	13 (5.78)	8 (3.5)	5 (2.6)	8 (4.2)	8 (4.2)	4 (4.12)	3 (3.09)	3 (0.09)
10	16 (1.9)	5 (0.61)	43 (5.2)	9 (1.1)^a^	6 (0.7)	32 (4.1)	12 (2.32)	-	24 (4.63)

Indication 1: AFD (Acute fetal distress) Test Chi square, test HB

^a^ p<0.01 between 2005–2007 and 2008–2010. Indication 2: CPD (Cephalopelvic disproportion) Indication 3: PET: Pre-eclampsia: Test Chi square, test HB

* p<0.01 between 2000–2004 and 2008–2010.

Cephalopelvic disproportion (groups 1 to 5) and acute fetal distress (groups 1 to 4b and group 10) are the main indications for CS. In the group 1 of Robson, the trend is increased overtime (P<0.01). In the group 7, the indication for CS was significantly reduced over time (P<0.01). In the other Robson groups, the rates remained unchanged. The third main indication for CS was pre-eclampsia. However, the rates are lower than for fetal distress in the groups 1 to 4b. In the groups, 8 and 10 of Robson the indication of CS due to PET was higher than that due to fetal distress. Interestingly, the indication for CS in cases of PET was reduced with time in the groups 1, 2a and 2b of Robson. In the other Robson groups, no differences were observed with time (Table 8).

**Table 8:** Trends in indications for caesarean section in the relevant categories of women in the Robson classification in Peru, 2000–2010.

Robson Group	2000–2004			2005–2007			2008–2010		
	Acute fetal distress	Pre-eclampsia	Cephalopelvic disproportion	Acute fetal distress	Pre-eclampsia	Cephalopelvic disproportion	Acute fetal distress	Pre-eclampsia	Cephalopelvic disproportion
	n (%)	n (%)	n (%)	n (%)	n (%)	n (%)	n (%)	n (%)	n (%)
1	1,810 (20.7)*	609 (6.97)*	2,657 (31.2)^a^	2,036 (21.4)	667 (7.01)	2,523 (27.4)	2,120 (22.9)	512 (5.54)	2,512 (29.0)
2a	202 (20.3)	129 (12.9)^a^	191 (19.3)	155 (15.4)	87 (8.64)	147 (14.6)	136 (20.1)	44 (6.52)	94 (14.0)
2b	1,508 (17.8)^b^	1,026 (12.2)*	2,087 (25.0)*	1,370 (16.1)	1,021 (12.0)	2,243 (26.7)	1,300 (16.6)	743 (9.50)	2,243 (29.1)
3	801 (22.2)	217 (6.01)	640 (18.0)*	910 (23.1)	266 (6.75)	671 (17.6)	850 (22.8)	211 (5.65)	732 (21.2)
4a	87 (28.5)	24 (7.87)	46 (15.8)	60 (20.7)	16 (5.52)	37 (12.8)	71 (25.7)	10 (3.62)	23 (8.39)
4b	789 (16.6)	491 (10.3)	520 (10.9)^a^	792 (15.2)	537 (10.3)	836 (16.1)	558 (14.7)	365 (9.60)	620 (16.5)
5	450 (4.53)^c^	317 (3.19)	1,397 (14.0)^a^	387 (3.94)	296 (3.01)	1,091 (11.2)	347 (3.67)	289 (3.06)	1,106 (12.1)
6	45 (1.38)	78 (2.39)	49 (1.51)	30 (1.09)	55 (2.01)	40 (1.48)	30 (1.41)	40 (1.87)	41 (1.97)
7	74 (2.82)*	76 (2.89)	65 (2.48)^b^	64 (2.41)	51 (1.92)	39 (1.49)	27 (1.20)	48 (2.13)	43 (1.96)
8	55 (2.47)	190 (8.54)	24 (1.13)	62 (3.01)	154 (7.49)	9 (0.45)	28 (1.75)	116 (7.23)	8 (0.54)
9	59 (2.98)	49 (2.47)	45 (2.28)	48 (2.58)	31 (1.67)	31 (1.69)	28 (1.91)	33 (2.25)	40 (2.85)
10	498 (10.3)	1,199 (24.7)	239 (5.03)^d^	509 (9.86)	1,279 (24.7)	181 (3.61)	481 (10.7)	1,045 (23.3)	198 (4.81)

Time trend

* p<0.01 2000–2004 vs2008–2010; 2005–2007 vs2008–2010

a: p<0.01:2000–2004 vs2005–2007;2000–2004 vs2008–2010.

b:2000–2004 vs2005–2007

c: p<0.01 2000–2004 vs 2008–2010

d: p<0.001: 2000–2004 vs 2005–2007; 2005–2007 vs 2008–2010

## Discussion

The 10-group classification system described by Robson was applied in the large database from the Peruvian perinatal Information system, which involved 549,681 deliveries. This database includes information of three different geographical regions in Peru characterized by differences in socio-economic status and in health facilities. For instance, in the coast were included three hospitals of category III, whereas none was included at high altitudes or the jungle of Peru.

In Peru, CS rates in 43 public hospitals have increased over a 10 year period from 25.5% in 2000 to 30.1% in 2010 well above 10–15 percent accepted as optimal rate for medically necessary cesarean delivery [3,12]. In the present study, we showed that this increase in CS rate was due to the contribution of the Robson groups 1, 3, 4a, 5, 7 and 10. Group 1 (nulliparous women with singleton cephalic full-term pregnancy in spontaneous labor), group seven (multiparas with a single breech pregnancy) and group 8 (multiple pregnancy) show the main increase overtime. During 2000–2004 the main relative contributions for CS (over 15%) were group 5, 1 and 2b respectively. This pattern was maintained during 2008–2010 being significant the increase for the group 1.

As in other published studies [15, 16] using the classification, the largest group in our obstetric populations is represented by the Robson group 3, which includes multiparous women with a singleton fetus in cephalic presentation. In our database, this represents 34.3% of the obstetric population, a similar value to the 32.3% observed in a Latin American survey [15]. In this group 3 is observed an overall rate of 5.73% of CS (2000–2010) with a trend to increase overtime, a value lower to that described in 2004–2005 for eight countries in LA (9.9%) [15]. It is expected these women are of low risk for CS. Another low risk group for CS is the group 1 (nulliparous with single cephalic pregnancy at term in spontaneous labor). We have demonstrated a value of CS rate of 14.0% during the 10-years period, which was lower to that observed in eight LA countries [15]. Both groups represent 27.5% of all CS.

In a tertiary hospital in Singapore between 2000 and 2010, groups 5 and groups 1 contributed mainly to the overall increase in CS rate from 19.9 to 29.6 per 100 births in the period of 2000–2010 [17]. In Peru, a similar pattern is observed. However, when estimating the main contributors to the CS rate in the Peruvian population, these were the groups 5 (18.9%) followed by group 1 (17.4%). Group 2b (nulliparous with planned cesarean section) has a relative contribution of 16.1% without changes overtime during the 10-years period. Fetal distress and preeclampsia could be important causes for CS, however in the present study the highest indication for CS due to fetal distress were in the groups 4a, 3, 2a and 1 ranging from 20.7% to 28.5% whereas indication for CS due to preeclampsia was higher in the group 10 (24.7%). These data suggest that reducing CS in groups 1, 2b, 3 and 5 the total CS rate may be significantly reduced in Peru. Group 1 contributed more than others to increase the overall CS rate between 2000 and 2010. This has also been observed in a previous study in Ireland [18]. According to our present data, there is no reason to increase CS in the group 1 since indication due to fetal distress is in the order of 20% for fetal distress and 6% for preeclampsia.

Although the 10-group classification system is used broadly, our data suggest that is better a subdivision of the groups 2 (nulliparous) and 4 (multipara without uterine scar). Data revealed different behaviors between both subgroups. In addition, analyzing outcomes by Robson group may allow a more action oriented comparisons between CS and vaginal delivery. In fact, the results of the present study showed that stillbirths and maternal mortality have significantly high ORs for CS than vaginal delivery in groups 1 and 3. Since groups, one and three are at term and in spontaneous labor is expected that all women belonging in these groups must to end in a vaginal delivery. However, when women of these groups are submitted to CS, a higher risk for stillbirths and for maternal mortality are observed.

Although data from stillbirths and maternal mortality were controlled for preeclampsia in the analysis, we have also studied the association between preeclampsia and CS in each Robson group. As expected, preeclampsia was strongly associated with CS in nine of the 10 Robson groups. This is because the first line of treatment for preeclampsia is CS delivery in Peru. Patients with a history of pre-eclampsia were 2.5 times more likely to have cesarean delivery (OR = 2.5; p<0.02) [19]. It is possible that cases of stillbirths and maternal mortality associated with CS could be also associated to preeclampsia [20]. Robson in 2013 suggested that satisfactory CS rate in the group 1 would be around 10% and in that for Group 3, the caesarean section rate should be no higher than 3%. Clearly, CS rates for these groups in the Peruvian database are above these proposed numbers [2].

The Robson Classification system applied to Peruvian population may guide us through groups in which not medically justified CS may be conducted; e.g. those belonging to the groups 1 and 3 comprising pregnancies at term with a singleton in cephalic position with spontaneous labor. The fact that trend of CS in groups one and 3 increases over time suggests that reduction in CS rates could be obtained by reduction of CS in these groups, particularly because CS represents higher costs than vaginal deliveries [21] but also because these groups under CS deliveries are also associated with maternal and perinatal adverse outcomes. Robson classification system has been applied broadly in high- and middle-income countries [10,14]. Only three studies have been published in low-income countries [22–24]. Peru is a low-income country where reducing maternal and perinatal morbidity and mortality is a priority for the Ministry of Health. Data obtained in the present study is important since women and neonates may be at higher risk of adverse outcomes due to unnecessary cesarean sections as those related to the group 1 of Robson. However, we must to recognize that the higher risk of adverse outcome associated with cesarean delivery could be influenced by the reason for which the cesarean section was indicated and not only for the CD per se.

In countries with low cesarean rates (>15%) an inverse association was observed with neonatal, and maternal mortality rates. In countries with high cesarean rates (>15%) no association was observed with infant or maternal mortality [25]. In contrast, our study, particularly in groups 1, 2 and 5 in which vaginal delivery should be the first choice, showed that OR for stillbirths and maternal mortality was significantly increased related to vaginal delivery.

Then, in accordance with other suggestions [26], this standardized classification should be used by Ministries of Health to monitor CS rates and to identify where interventions should be done to reduce the unjustified high CS rates. This classification should be used in all hospitals to be able to monitor of its obstetric practices to be sure if a CS was the right choice.

A unique feature of Perinatal Information System (SIP in Spanish) using in the study is that antenatal data are linked with birth outcome data. Therefore, SIP data is collected prospectively by clinicians in a hard-copy form. Information in the hard-copy SIP form was later entered by clerks into a database using SIP software.

In conclusion, data from Peru showed that CS rates increased over time because of increased CS in groups with spontaneous labor (groups 1 and 3) and in-group of multipara with a scarred uterus and with a single cephalic term pregnancy. Robson classification systems allow us to identify that groups 1 and 3 had increased OR for stillbirths, a maternal mortality by cesarean section than with vaginal delivery. Women with previous cesarean section constitute the most important determinant of overall cesarean section rates. However, rate of postpartum hemorrhage double from 2000–2004 to 2008–2010. In summary, use of Robson classification becomes in useful tool to monitoring cesarean section in low human development index countries.
